# Design of a Gough–Stewart Platform Based on Visual Servoing Controller

**DOI:** 10.3390/s22072523

**Published:** 2022-03-25

**Authors:** Minglei Zhu, Cong Huang, Shijie Song, Dawei Gong

**Affiliations:** School of Mechanical and Electrical Engineering, University of Electronic Science and Technology of China, Chengdu 611731, China; minglei.zhu@uestc.edu.cn (M.Z.); 202121040210@std.uestc.edu.cn (C.H.); 201721080102@std.uestc.edu.cn (S.S.)

**Keywords:** parallel robot, image-based visual servoing, optimal design, Gough–Stewart platform, controller singularity, hidden robot

## Abstract

Designing a robot with the best accuracy is always an attractive research direction in the robotics community. In order to create a Gough–Stewart platform with guaranteed accuracy performance for a dedicated controller, this paper describes a novel advanced optimal design methodology: control-based design methodology. This advanced optimal design method considers the controller positioning accuracy in the design process for getting the optimal geometric parameters of the robot. In this paper, three types of visual servoing controllers are applied to control the motions of the Gough–Stewart platform: leg-direction-based visual servoing, line-based visual servoing, and image moment visual servoing. Depending on these controllers, the positioning error models considering the camera observation error together with the controller singularities are analyzed. In the next step, the optimization problems are formulated in order to get the optimal geometric parameters of the robot and the placement of the camera for the Gough–Stewart platform for each type of controller. Then, we perform co-simulations on the three optimized Gough–Stewart platforms in order to test the positioning accuracy and the robustness with respect to the manufacturing errors. It turns out that the optimal control-based design methodology helps get both the optimum design parameters of the robot and the performance of the controller {robot + dedicated controller}.

## 1. Introduction

Parallel robots are becoming more and more attractive due to their better performances compared with classical serial robots in terms of high speed and acceleration, payload, stiffness, and accuracy [1]. Nevertheless, the traditional control of parallel robots is always troublesome because of the high non-linear input/output relations.

It can be found in [2] that a large number of researches have focused on the control of parallel robots. Generally, the only way to ensure high accuracy of a parallel robot is to get the robot model as detailed as possible for the model-based controller [3]. However, due to several factors such as errors from manufacturing and robot assembly, even detailed models still suffer from the problem of inaccuracy in practice. Therefore, more and more researches are currently focusing on finding an alternative controller to sidestep the complex kinematic architecture of the robot and to reach a better positioning accuracy performance compared with the classical model-based controllers. The sensor-based controller is an efficient method that estimates the pose of the end-effector with external sensors [4,5,6]. Visual servoing is a sensor-based controller, which takes one or several cameras as external sensors and closes the control loop by the vision information obtained from the camera. Visual servoing can be classified into two main groups: position-based visual servoing (PBVS) and image-based visual servoing (IBVS). PBVS directly controls the pose of the target with respect to the camera in Cartesian space [7]. Image-based visual servoing aims at minimizing the errors between current image features and desired image features directly in image space. It is more robust with the calibration errors compared with PBVS and can make sure that the target is always in the image plane so that we do not lose track when servoing. Therefore, we propose to apply IBVS as the external sensor-based controller in this paper. A large number of researches focused on controlling parallel robots with IBVS with the development of the image processing and image acquisition technology [5,8,9,10,11,12,13,14]. It has been proven that the end-effector pose can be estimated effectively throughout the direct observation by vision [15] or the indirect observation [14,16,17,18]. In addition, the choices of image features applied in visual servoing of parallel robots are numerous, such as image moments [19,20] when the camera can observe the end-effector directly or the observation of robot legs when directly observing the end-effector is difficult to realize (such as the machine tool) [9].

When the vision-based controller is applied to control parallel robots, the positioning accuracy is one of the most important internal performances and the positioning accuracy comes from the error of observation of the image features [21]. The types and numbers of cameras that are used, together with the kinds of image features, all have an influence on the observation error [22]. In addition, the geometric parameters of robots and the camera position also affect the positioning accuracy since they change the interaction models, which leads to effects on the positioning accuracy [23,24]. One problem that should be mentioned is that the mapping between the image feature space and the Cartesian space is not free of singularities [25]. The existence of the singularity of the interaction model has a great influence on the accuracy performance of the parallel robot [26]. In conclusion, in order to ensure the best accuracy performance for the pair {robot +controller} throughout its workspace, the robot geometric parameters and camera position should be optimized in advance.

The optimal design methodology of the robot aims at getting the optimal design geometric parameters of the robot to minimize a given objective under constraints. In [27], when visual servoing is applied to the control of parallel robots, the controller singularity and the internal performance (especially the positioning accuracy) should be taken into account in advance. In addition, the visual servoing controller is never considered in the optimal design process before. Therefore, in this work, the “control-based design” methodology considering the controller performance is developed and the positioning accuracy, together with the controller singularity of the corresponding controllers will be taken into account during the robot design process in order to get the optimal geometric parameters of a Gough–Stewart platform for a dedicated controller with the best performance of accuracy and to avoid the instability issues that appear in the control process. In this case, three types of vision-based controllers will be considered:

- Leg-direction-based visual servoing (LegBVS) [20];
- Line-based visual servoing (LineBVS) [28];
- Image-moment-based visual servoing (IMVS) of a feature mounted on the platform [19].

To the best of our knowledge, this is the first time that we design a spatial 6 DOF parallel robot with the optimal control-based design methodology. In addition, this is the first time that the topological optimization is applied in the image moment visual servoing controller design.

This paper is organized as follows: Section 2 presents the robot architecture, design requirements, and the specifications of visual servoing controllers. The concept of visual servoing applied for controlling the Gough–Stewart platform is reviewed in Section 3. In Section 4, the controller accuracy performance (the error model relating the error from the camera observation to the positioning error of the robot ) and controller singularities, which lead to the instability of the robot, are discussed. Optimal design procedure based on the visual servoing controllers is introduced and solved in Section 5. Then, in Section 6, the co-simulation between Simulink and ADAMS with the result analysis are described. Finally, some conclusions are drawn in Section 7.

## 2. Robot Architecture and Specification

**Figure 1:**
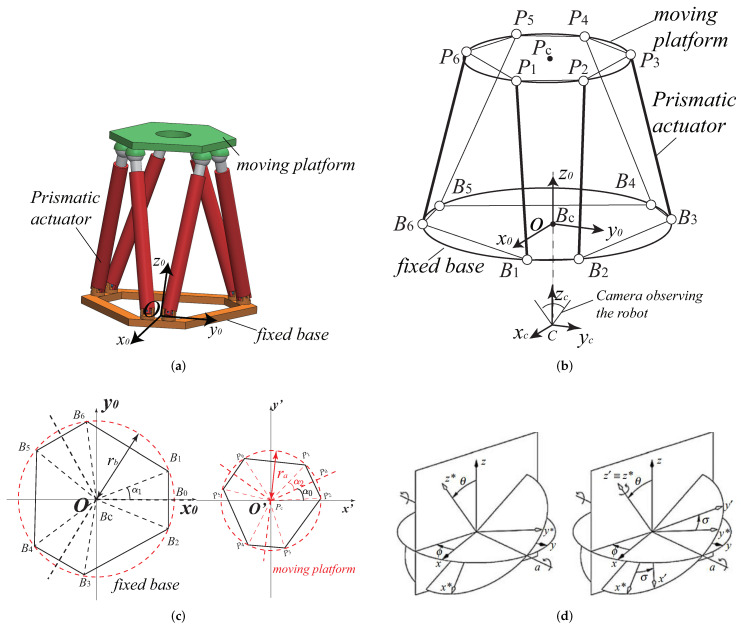
Schematics of the Gough–Stewart platform. (**a**) CAD of the Gough–Stewart platform with its regular dexterous workspace; (**b**) Schematics of the Gough–Stewart platform architecture; (**c**) Gough–Stewart platform geometric design parameters; (**d**) Tilt and torsion angles.

In this paper, we optimize the geometry of the Gough–Stewart platform with visual servoing in order to get excellent performance of the pair {robot+controller}. The Gough–Stewart platform, also called a hexapod, is a parallel robot with 6 degrees of freedom (DOF): the moving platform of the Gough–Stewart platform translates along the three axes of the space and rotates around the three axes of the space with respect to the fixed base [29]. The Gough–Stewart platform designed in this chapter is a 6-UPS robot (Figure 1a). The moving platform of the robot is linked to the fixed base by 6 individual chains $B_{i}P_{i}\;\left(i=1\cdots 6\right)$. The connection of the chains with the base is a U joint located at $B_{i}\;\left(i=1\cdots 6\right)$, the chains are attached to the end-effector by a S joint located at $P_{i}\;\left(i=1\cdots 6\right)$ and the prismatic actuator allows the change of the lengths of the links $B_{i}P_{i}\;\left(i=1\cdots 6\right)$ (Figure 1b).

The base and the moving platform of the considered Gough–Stewart platform are symmetric hexagons (Figure 1c). The radius of the circumcircle of the base is $r_{b}$, and the radius of the circumcircle of the moving platform is $r_{a}$. The angle $∠B_{1}B_{c}B_{2}=2\alpha_{1}$, the angle $∠P_{1}P_{c}P_{2}=2\alpha_{2}$ and the angle $∠x^{\prime }P_{c}P_{0}=\alpha_{0}$ (Figure 1c).

The complete workspace of the Gough–Stewart platform is a six-dimensional space. We should consider both its 3D location and the orientation of the moving platform. The definition of its orientation workspace is based on the Tilt and Torsion (T&T) angles proposed in [30]. The tilt and torsion angles are defined in two stages: a tilt and a torsion, In the first stage, as illustrated in Figure 1d, the frame *i* first rotates about the base $z_{i}$-axis by an angle ϕ, then about the $y_{i}$-axis by an angle θ, then about the $z_{j}$-axis by an angle −ϕ, and finally about the new $z_{k}$-axis by an angle σ. The expression of the rotation matrix of the T&T angles can be found in [30]. With the T&T angles, a novel 3D workspace subset named maximum tilt workspace was developed in [31]. This workspace measure is defined as the set of positions that the center of the moving platform can attain with any direction of its *z*-axis making a tilt angle limited by a given value. Therefore. the orientation workspace of the Gough–Stewart platform can be kept to be symmetrical. Then, the configuration of the Gough–Stewart platform can be defined by the vector $x=[x_{t},y_{t},z_{t},\phi ,\theta ,\sigma ]$ while $\left[x_{t},y_{t},z_{t}\right]$ represents the 3D location of the center of the moving platform and [ϕ,θ,σ] defines the T&T angles.

The requirements that must be achieved by the Gough–Stewart platform in this case are given in Table 1. They have been fixed after discussion with some of our industrial partners. First of all, the maximum tilt workspace of the Gough–Stewart platform should cover a cube of side length $l_{0}\geq 100$ mm and the range of T&T angles being ϕ∈(−π,π], θ∈[0,π/12], and σ∈[0,π/12]. In this workspace, several performances should be guaranteed. Thus, this cube will be called the regular dexterous workspace (RDW) of the robot [1].

Additionally, considering the reality (gain of place), the footprint of the robot must be as small as possible.

The Gough–Stewart platform optimized ought to satisfy all the following geometric and kinematic constraints throughout the RDW:

- The RDW should be free of singularity (both of the Gough–Stewart platform and the visual servoing controllers applied in this case);
- The robot positioning error should be lower than 1 mm;
- The robot orientation error should be lower than 0.01 rad;
- Some distances are constrained in order to avoid collisions or to have unpractical designs: the distance $r_{b}$ between the origin of the base frame *O* and the U joint position $B_{i}$, the distance $r_{a}$ between the origin of the platform frame $P_{c}$ and the S joint $P_{i}$, the radius of the prismatic actuator’s $B_{i}P_{i}$ cross-section denoted as $R^{\prime }$ and, finally, the camera frame location (Figure 1b). These constraints will be further detailed in Section 5.

In order to get the desired 1 mm of positioning accuracy and 0.01 rad orientation accuracy specified in Table 1, we propose to apply visual servoing approaches. A single camera is chosen to be the external sensor and is mounted onto the ground in order to control the motions of the Gough–Stewart platform. The resolution of the camera is 1920×1200 pixels and the focal length is 10 mm). The best way is to observe some image features attached to the moving platform directly with the camera. However, in some cases, it is difficult to observe the end-effector, such as the milling operations. Alternative features proposed in [20] are the cylindrical legs of the robot’s prismatic actuators. Therefore, in this case, three types of classical visual servoing approaches (LegBVS [20], LineBVS [28], and IMVS [19]) will be tested.

The two first controllers take the image features extracted from the observation of robot legs, while the last one will be used to observe the platform directly. The optimal design parameters of the Gough–Stewart platform for each type of controller will be found and based on the analysis of the obtained results, the best pair {robot + controller} will be determined.

In addition, several comments should be illustrated here. First, the dynamic criterion is not mentioned in these specifications. In fact, for the visual servoing, high-speed motion is not the purpose, except for a few specific scenarios [32,33]. Therefore, only the geometry and kinematics performance of the robot will be considered. Besides, a repeatability of 1 mm and orientation accuracy of 0.01 rad could also be obtained by a standard encoder-based controller. However, this paper does not aim to prove that visual servoing gets a better accuracy performance compared with standard encoder-based control. This paper aims to prove that in the condition of controlling a robot with visual servoing (or any other types of sensor-based controllers), in order to obtain the guaranteed accuracy, it is essential to optimize the robot and the controller at the same time in the design process.

In the next section, some brief recalls on visual servoing will be given in front of presenting the optimization problem formulation.

## 3. Recalls on Visual Servoing

In this section, a simple review on visual servoing is presented. Then, we provide some recalls on three considered approaches in particular [19,20,28].

### 3.1. Basics of Image-Based Visual Servoing

Image-based visual servoing is an external sensor-based controller which uses the so-called interaction matrix L [5] to transform the twist ${}^{c}\tau_{c}$ between the camera and the scene (in what follows, the superscript “*c*” denotes the camera frame), to the time derivative $\dot{s}$ of the vector s of the visual primitives observed from the camera through the relationship:

(1) $$\dot{s}=L\left(s,x\right){\;}^{c}\tau_{c}$$

the components of L are highly nonlinear and are a function of the image features s and the robot end-effector configuration in Cartesian space x.

Based on (1),we can build a simple visual servoing error model:

(2) Δs=L(s,x)Δx

where Δs represents a small error from the camera observation and Δx is the corresponding positioning error of the robot end-effector in Cartesian space. As mentioned above, the matrix L is a matrix whose components are nonlinear functions depending of the variables s and x. Therefore, the matrix L may meet some singularities. Positioning error models and singularities of the visual servoings [19,20,28] will be further detailed in Section 4. Now, let us provide some recalls about the features observed in the three different types of controllers [19,20,28].

In addition, based on the kinematic relationship, one classical controller that takes the image feature s as the feedback can be proposed:

(3) $${}^{c}\tau_{c}=-\lambda L^{+}e$$

in which the vector e stacks the error between the desired image feature $s^{*}$ and the current one $e=s-s^{*}$, $L^{+}$ is the pseudo-inverse of the matrix L, and λ is a positive constant.

This expression can be transformed into a controller for the joint velocities:

(4) $$\dot{q}=-\lambda J_{pinv}L^{+}e$$

where $J_{pinv}$ is the inverse Jacobian matrix of the robot linking the end-effector twist to the actuator velocities, i.e., $J_{pinv}{\;}^{c}\tau_{c}=\dot{q}$.

### 3.2. Recalls on Leg-Direction-Based Visual Servoing and Line-Based Visual Servoing

**Figure 2:**
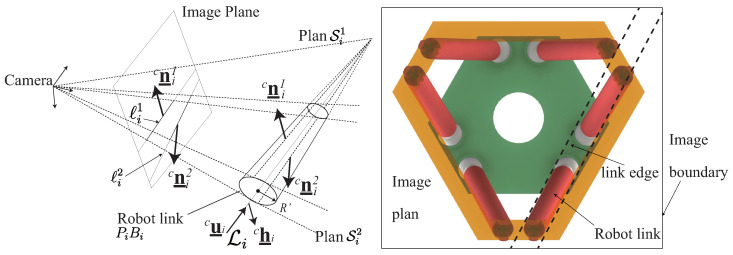
Projection of a cylinder in the image plane and the image from the camera observation.

The legs of parallel robots are usually designed with slim and cylindrical rods, then the feature that can be extracted from the observation of the legs are their directions ${}^{c}{\underline{u}}_{i}$ (Figure 2) [20] and the line $L_{i}$ passing through robot link cylinder axis *i* expressed by its Plücker coordinates ${(}^{c}{\underline{u}}_{i}{,}^{c}{\underline{h}}_{i})$ (see definition in [11] and Figure 2).

For Leg-direction-based visual servoing, we can always find the relationship between the twist of robot end-effector ${}^{c}\tau_{c}$ and its leg direction velocity by:

(5) $${}^{c}{\underline{\dot{u}}}_{i}=M_{ui}^{T}{\;}^{c}\tau_{c}$$

where $M_{ui}^{T}$ is the interaction matrix for the leg *i*.

For Line-based visual servoing, the kinematic model aims at finding the relationship between the time variation of the Plücker coordinates ${(}^{c}{\underline{u}}_{i}{,}^{c}{\underline{h}}_{i})$ of the robot legs and the twist of its platform [9]:

(6) $$[\begin{array}{c} {}^{c}{\underline{\dot{u}}}_{i}^{T}\\ {}^{c}{\underline{\dot{h}}}_{i}^{T} \end{array}]=M_{uhi}^{T}{}^{c}\tau_{c}$$

$M_{uhi}^{T}$ is the interaction matrix for the leg *i* for this type of observation.

In image plane, the contour of these cylindrical links are projected into lines $\ell_{i}^{\left(k\right)}$ (k=1,2) defined as the intersections of the image plane and the plane $S_{i}^{\left(k\right)}$ with normal ${}^{c}n_{i}^{\left(k\right)}$ lying on the camera frame origin *C* and the observed cylinder (see Figure 2). With the coordinates of the intersection points between the lines $\ell_{i}^{\left(k\right)}$ (k=1,2) and the image plane boundary, together with the position of the camera, we can get the vector ${}^{c}n_{i}^{\left(k\right)}$ of the plan $S_{i}^{\left(k\right)}$. Therefore, for each cylindrical robot leg, the normal vectors ${}^{c}n_{i}^{\left(k\right)}$ are information that we can extract from the camera observation. If *n* cylinders are observed, the vector s of the observed features is defined as: $s={\left[{}^{c}n_{1}^{(1)T}\;\;{}^{c}n_{1}^{(2)T}\ldots {}^{c}n_{n}^{(1)T}\;\;{}^{c}n_{n}^{(2)T}\right]}^{T}$. Moreover, as we see from Figure 2, it should be mentioned that only observing part of one robot leg (the entire view of the leg is not essential) can get the intersection points of its edges and the image plan boundary.

Therefore, for leg-direction-based visual servoing, we can get the relationship between the time variation of ${}^{c}{\underline{u}}_{i}$ and the derivative of image features ${\left[{}^{c}n_{i}^{(1)T}\;\;{}^{c}n_{i}^{(2)T}\right]}^{T}$ with respect to time with

(7) $${}^{c}{\dot{\underline{u}}}_{i}=J_{ui}[\begin{array}{c} {}^{c}{\dot{\underline{n}}}_{i}^{1}\\ {}^{c}{\dot{\underline{n}}}_{i}^{2} \end{array}]$$

$J_{ui}$ transforms the derivative with respect to time of $\left({}^{c}{\underline{n}}_{i}^{1},{}^{c}{\underline{n}}_{i}^{2}\right)$ into the leg orientation velocities [28].

For Line-based visual servoing, similar to Leg-direction-based visual servoing, we obtain:

(8) $${}^{c}{\dot{\underline{u}}}_{i}=J_{ui}[\begin{array}{c} {}^{c}{\dot{\underline{n}}}_{i}^{1}\\ {}^{c}{\dot{\underline{n}}}_{i}^{2} \end{array}],{}^{c}{\dot{\underline{h}}}_{i}=J_{hi}[\begin{array}{c} {}^{c}{\dot{\underline{n}}}_{i}^{1}\\ {}^{c}{\dot{\underline{n}}}_{i}^{2} \end{array}]$$

where $J_{ui}$ and $J_{hi}$ transform the time derivative of $\left({}^{c}{\underline{n}}_{i}^{1},{}^{c}{\underline{n}}_{i}^{2}\right)$ into the vector velocities of $\left({}^{c}{\underline{n}}_{i},{}^{c}{\underline{h}}_{i}\right)$.

To fully control the six DOF of the Gough–Stewart platform, observing a minimum of three independent legs is necessary. Therefore, when using LegBVS, we obtain the end-effector twist ${}^{c}\tau_{c}$ with:

(9) $$M_{u}^{T}{\;}^{c}\tau_{c}=J_{u}{\;}^{c}\underline{\dot{n}}$$

The matrix $M_{u}^{T}$ can be obtained by stacking the matrices of $M_{ui}^{T}$ of *k* legs (k=3,⋯,6) (the way of getting the interaction matrix $M_{u}^{T}$ is presented in Appendix A). $J_{u}$ is a block-diagonal matrix containing the matrix $J_{ui}$. By using the pseudo-inverse $M_{u}^{T+}$ of the matrix $M_{u}^{T}$, we have:

(10) $${}^{c}\tau_{c}=M_{u}^{T+}J_{u}{}^{c}\underline{\dot{n}}=L_{u}^{T+}{\;}^{c}\underline{\dot{n}}$$

In the condition of Line-based visual servoing, the end-effector twist ${}^{c}\tau_{c}$ can be obtained from the function:

(11) $$M_{uh}^{T}{\;}^{c}\tau_{c}=[\begin{array}{c} J_{u}\\ J_{h} \end{array}]\;{}^{c}\underline{\dot{n}}$$

The matrix $M_{uh}^{T}$ is obtained by stacking the matrices $M_{uhi}^{T}$ of *k* legs (k=3,⋯,6) (the way of getting the interaction matrix $M_{uh}^{T}$ is presented in Appendix A), $J_{u}$, $J_{h}$ are block-diagonal matrices containing the matrices $J_{ui}$, $J_{hi}$. Then, by using the pseudo-inverse $M_{uh}^{T+}$ of the matrix $M_{uh}^{T}$, we have:

(12) $${}^{c}\tau_{c}=M_{uh}^{T+}[\begin{array}{c} J_{u}\\ J_{h} \end{array}]{}^{c}\underline{\dot{n}}=L_{uh}^{T+}{\;}^{c}\underline{\dot{n}}$$

### 3.3. Image Moment Visual Servoing

**Figure 3:**
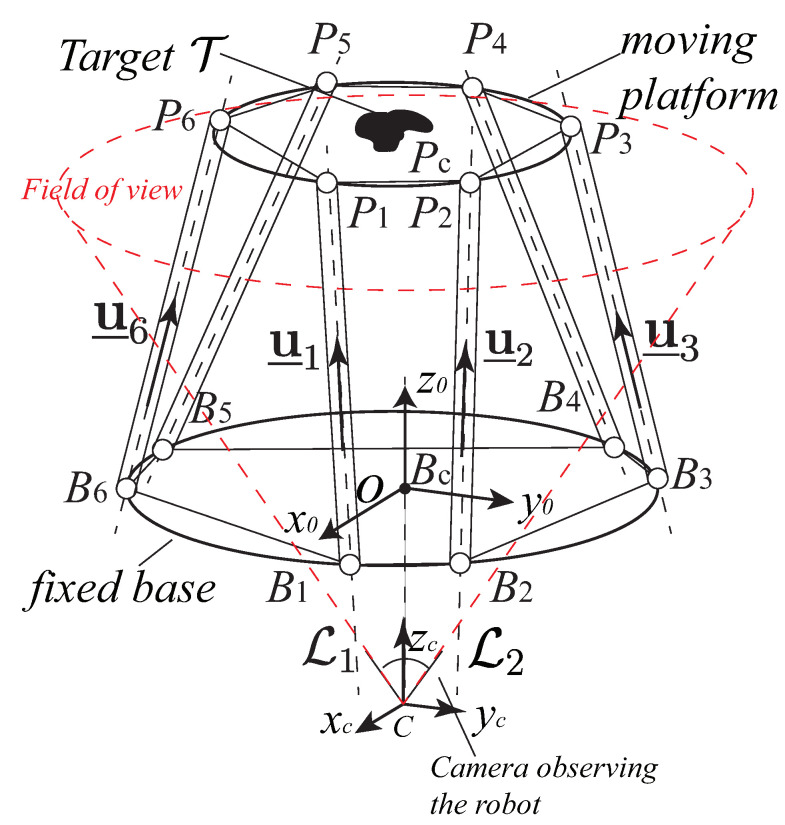
A camera observing the robot legs.

IMVS is different from the previous ones. It is an approach based on the observation of a target T mounted on the moving platform of the robot (Figure 3). The image moments can be extracted from the image plane through the observation of the camera [19]. The target T can be a dense object defined by a set of closed contours or a discrete set of *m* image points [34]. For the target T, we denote U the projection of the target in image plane. Then we can compute the image moment of U: the moment $m_{wt}$ of order w+t is defined by:

(13) $$m_{wt}={\;\int \int \;}_{U}^{}u^{w}v^{t}dxdy$$

where *u* and *v* are the coordinates in the image plane of any point belonging to the surface U. The interaction matrix associated with any moment is provided in [19].

**Figure 4:**
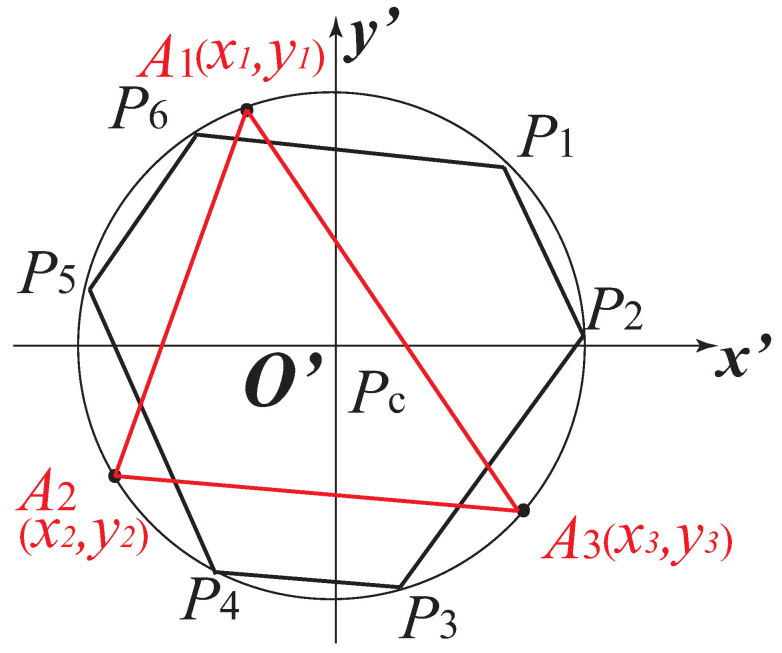
Discrete model composed of three points for image moment visual servoing.

For a Gough–Stewart platform with six DOFs, a set of six independent moments should be selected as the image features. In this work, T is set to be a discrete model composed of three points ($A_{1}$, $A_{2}$, $A_{3}$) (Figure 4). The selection of the proper image features is always a complex problem. We especially need to find six combinations of moments to control the six DOFs of the robot. The best selection of the visual servoing features is that they can be used to design a decoupled control scheme. That is try to associate each DOF to be controlled with only one visual feature. This can provide a large domain of convergence, a good behavior of the visual features, and an adequate camera trajectory. However, until now, no one found such a combination of image moments. In this paper, the objective is to obtain a sparse interaction matrix that changes slowly around the desired position and the selection of image moments is the same as it was in [34]. It has been proven that the coordinates $x_{g}$, $y_{g}$ of the center of gravity, and the area $a=m_{00}$ of the object are the classical image features are enough to control the three translational DOFs. In addition, in order to control the rotational DOF, we need to use the object orientation α and two moments $c_{1}$ and $c_{2}$ (see definitions in [34]). $c_{1}$ and $c_{2}$ have been proven to be invariant to translation and 2-D rotation. In conclusion, for image moment visual servoing applied in this case, the image feature is $m={\left[x_{g}\;y_{g}\;a\;\alpha \;c_{1}\;c_{2}\right]}^{T}$. Then we have:

(14) $$\dot{m}=L_{m}{\;}^{c}\tau_{c}$$

where $\dot{m}={\left[{\dot{x}}_{g}\;{\dot{y}}_{g}\;\dot{a}\;\dot{\alpha }\;{\dot{c}}_{1}\;{\dot{c}}_{2}\right]}^{T}$ are the time derivatives of six image features observed. $L_{m}={\left[L_{x_{g}}\;L_{y_{g}}\;L_{a}\;L_{\alpha }\;L_{c_{1}}\;L_{c_{2}}\right]}^{T}$ is the interaction matrix related to the set of image moments [19,34].

In the model for estimating the robot platform configuration based on the image features $m={\left[x_{g}\;\;y_{g}\;a\;\alpha \;c_{1}\;c_{2}\right]}^{T}$, the coordinates of the three points ($A_{1}$, $A_{2}$, $A_{3}$) (Figure 4) are involved, as well as the camera pose. The value of these parameters will be optimized later during the design optimization process.

It should be noticed that, despite the fact that there is no explicit appearance of the robot geometric parameters in the interaction model of image moment visual servoing controller, they still have an influence on its performance: the location of the robot workspace is defined by the robot geometric parameters. If the distance between the workspace and camera location is long, the accuracy performance will be worse than if the workspace is closer to the camera location. Accordingly, we still need to optimize the robot geometric parameters in order to optimize the overall robot accuracy.

In the next section, we deal with the computation of some performance indices of the visual servoing controller.

## 4. Controller Performance

Concerning the requirements of positioning accuracy for the robot design, two types of controller performance will be defined and considered:

- The presence (or even proximity) controller singularities, the singularities of the interaction matrices impact both the positioning accuracy and the controller stability [4];
- The positioning error comes from the camera observation error and the interaction model of the corresponding visual servoing controller.

Then, in this section, singularities of the corresponding controllers and the positioning error models are described.

### 4.1. Controller Singularities

It was defined in [35] that the rank deficiency of the interaction matrix L leads to the visual servoing controller singularity. In this section, based on the study of the controllers defined in Section 3, we show the conditions of rank deficiency of the corresponding interaction matrices.

#### 4.1.1. Leg-Based Visual Servoing Singularities

**Figure 5:**
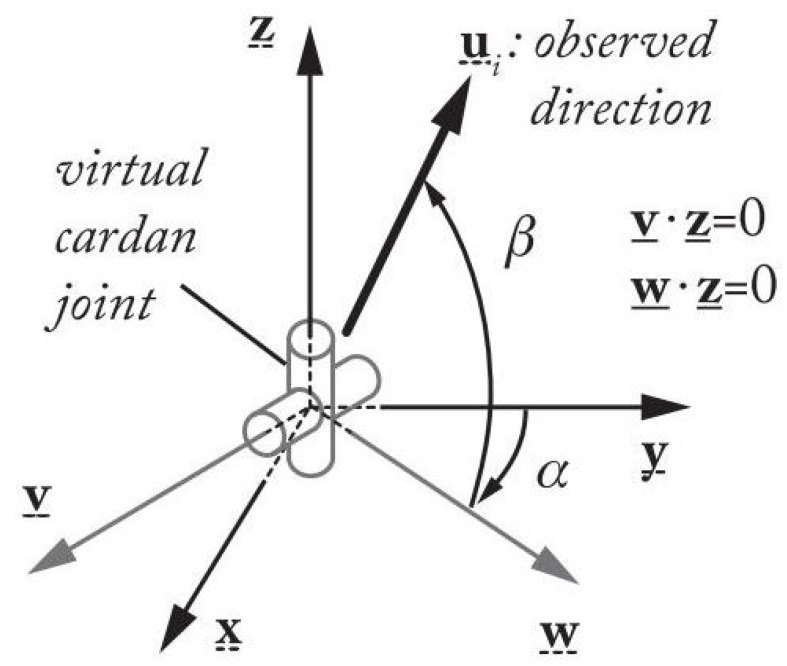
A unit vector ${\underline{u}}_{i}$ in space and its parameterization.

The singularity problem of the mapping between the space of the observed image features and the Cartesian space has a great influence on the accuracy of visual servoing. Thanks to the work of [36], a tool named “Hidden robot” was developed in order to simplify the study of the controller singularity problem when visual servoing is applied to the control of the parallel robot. It reduces the study of the complex singularities of the interaction matrix to the study of the singularities of the virtual parallel robot hidden in the controller. The main idea of the “Hidden robot” is to find the virtual actuators that correspond to the observation image features. For example, when we apply the Leg-direction-based visual servoing to control the Gough–Stewart platform, we choose the unit vector ${\underline{u}}_{i}$ as the image feature. The unit vector in space can be parameterized by two independent coordinates (see Figure 5) that can be the angles defined by the U joint rotations. Therefore, the displacement of the U joint can be measured by the vector ${\underline{u}}_{i}$. As a result, the U joint is the virtual actuator (in another way, the “hidden robot”) of the vector ${\underline{u}}_{i}$. From (4), we see that the visual servoing can meet numerical issues if the matrix $L^{T}$ is rank deficient and a null error vector e leads to a non-null platform twist ${}^{c}\tau_{c}$; or the matrix $L^{T+}$ is rank deficient and the controller may meet a local minimum, which means that the error e is not zero but the twist ${}^{c}\tau_{c}$ is zero. The interaction matrix L involved in the controller gives the value of $\dot{s}$ as a function of ${}^{c}\tau_{c}$. Therefore, $L^{T}$ can be seen as the inverse Jacobian matrix of the hidden robot (moreover, $H^{+}$ is the hidden robot Jacobian matrix). Then, $L^{T}$ is rank deficient only when the corresponding hidden robot comes to Type 2 singularity loci and $L^{T+}$ is rank deficient only when the corresponding hidden robot comes to Type 1 singularity loci. Therefore, the hidden robot helps simplify the analysis of the interaction matrix singularity by reducing this problem to the singularity analysis of a new robot.

In [37], the problem of LegBVS controller singularities for the control of the Gough–Stewart platform has been detailed presented. The Gough–Stewart platform consists of six $U\underline{P}S$ legs.The corresponding hidden robot of the $U\underline{P}S$ leg is made of $\underline{U}PS$ legs. Since $\underline{U}PS$ legs have 2 degrees of actuation, only three legs to be observed are enough to fully control the Gough–Stewart platform when using leg direction observation [36].

**Figure 6:**
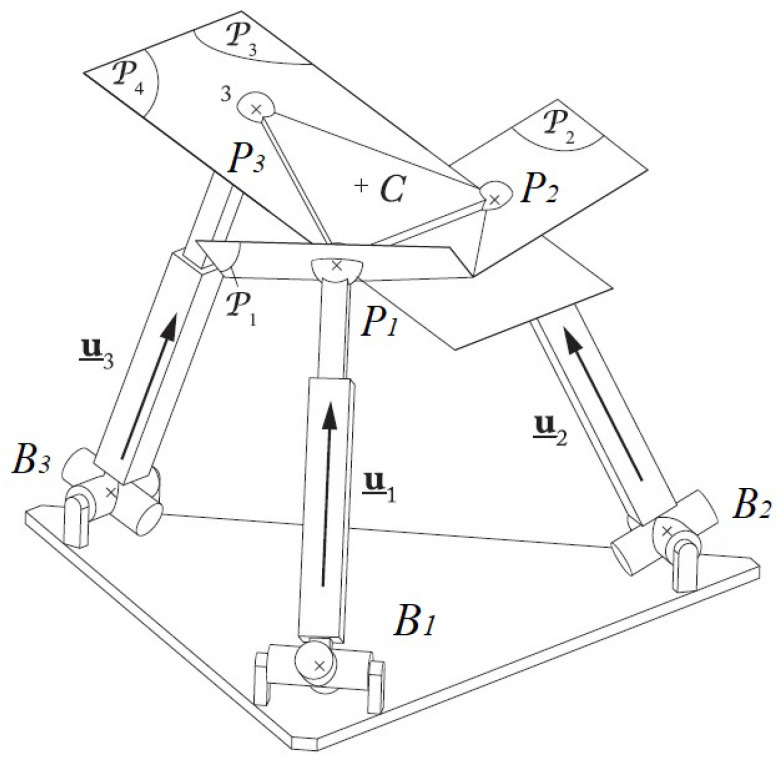
Example of a Type 2 singularity for a 3-$\underline{U}PS$ robot: the platform gets an uncontrollable rotation around $P_{1}P_{2}$ [37].

The singular configurations of 3-$\underline{U}PS$-like robots have been deeply studied in [38,39]. Type 2 singularities appear when the planes $P_{1},P_{2},P_{3}$ (whose normal directions are defined by the vectors ${\underline{u}}_{1}$, ${\underline{u}}_{2}$, ${\underline{u}}_{3}$ and the plane $P_{4}$ (passing through the points $P_{1},P_{2},P_{3}$ in Figure 6) intersect in one point (which can be at infinity) (Figure 6).

Singularities of LineBVS applied to the control of the Gough–Stewart platform have never been studied before. The concept of the hidden robot is to find what kind of virtual actuators correspond to the features of observation applied in visual servoing. For LineBVS, we take the Plücker coordinates of a line $L_{i}$ as the image feature to be observed and it can be defined from the fact that a 3D point and a 3D orientation define a unique 3D line [11]. Therefore, we should find the virtual actuators corresponding to the 3D line $L_{i}$.

**Figure 7:**
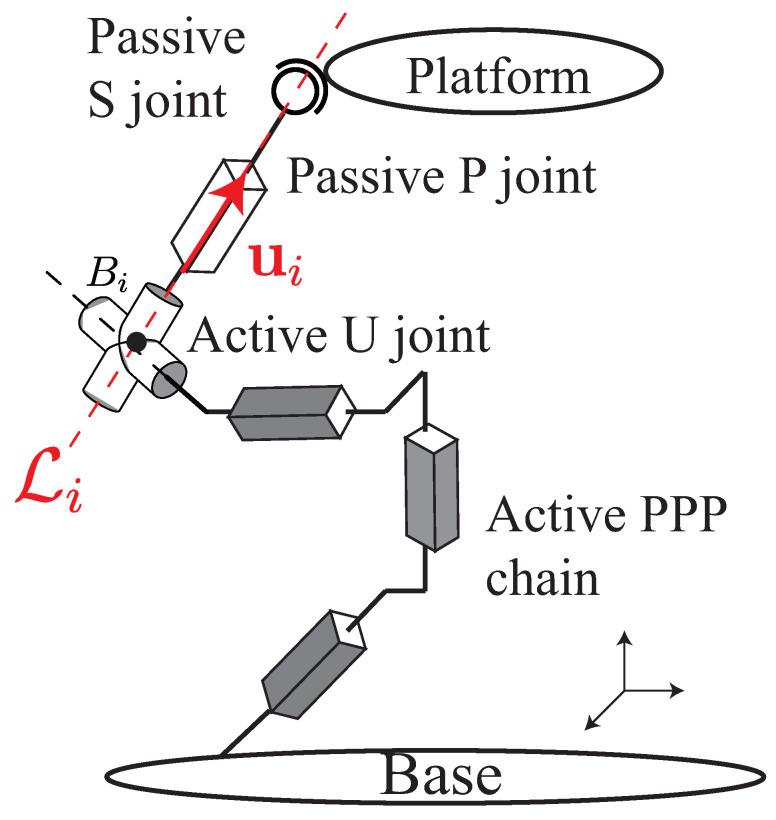
Corresponding hidden robot leg when the line $L_{i}$ in space is observed.

As we see from Figure 7, $B_{i}$ is the 3D point and ${\underline{u}}_{i}$ the unit vector, $L_{i}$ (i=1,2,⋯,6) is the 3D line they define. The active $\underline{U}$ joint in space is the virtual actuator that makes the vector ${\underline{u}}_{i}$ move. In general, the actuated PPP chain should be added on the preceding leg links so that the point $B_{i}$ can move in space. Therefore, for a $U\underline{P}S$ leg, its corresponding hidden robot when using line-based visual servoing is a $\underline{PPP}\underline{U}PS$ leg (Figure 7). However, in the case of a Gough–Stewart platform, all the *U* joints are fixed on the base, which means that the points $B_{i}$ are fixed in space. Then the actuated PPP chain is no longer needed and the 3D lines $L_{i}$ passing through the robot links can be defined only by the vectors ${\underline{u}}_{i}$. Therefore, the corresponding hidden robot of the Gough–Stewart platform is the same as the hidden robot when applying leg-direction-based visual servoing, the $3-\underline{U}PS$ robot, which means that these two visual servoing controllers share the same conditions of controller singularities. Then, we suppose that in terms of controller performances, LegBVS and LineBVS are the same (which will be proven in the following Section).

#### 4.1.2. Image Moment Visual Servoing Singularities

For IMVS, the controller singularity appears when the matrix $L_{m}$ is rank deficient. The expression of the matrix $L_{m}$ is rather complex and it is difficult to find the condition of rank deficient analytically. We should define a criterion of “proximity” to controller singularities. A list of indices that could be adapted in the analysis of robot singularity was presented in [40]. In this case, we take the inverse conditioning of the interaction matrix as the index of the controller singularity to estimate the numerical stability of the interaction matrix $L_{m}$.

### 4.2. Positioning Accuracy Model

#### 4.2.1. Observation Errors in the Leg-Based Visual Servoing

**Figure 8:**
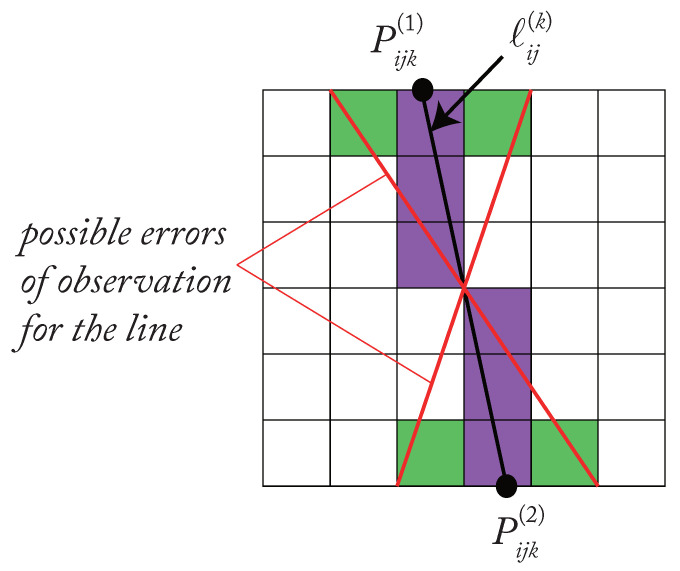
Error for the observation of a line.

The positioning error models when observing the robot links in the Leg-based visual servoing approaches have been detailed and presented in [22,24]. The positioning error comes from the camera observation error of image features (For LegBVS, the features are the leg directions, for LineBVS, the features are the leg Plücker coordinates). When we use the camera to observe the robot links, the link edges are projected into the image plane into lines $\ell_{ij}^{\left(k\right)}$ (Figure 3), which are then pixelized (Figure 8). We suppose that the error of estimation of the lines $\ell_{i}^{\left(k\right)}$ is due to a random shift of ±0.5 px in the pixels corresponding to the intersections of the $\ell_{i}^{\left(k\right)}$ with the image plane boundary (points $P_{ik}^{\left(1\right)}$ and $P_{ik}^{\left(2\right)}$ in Figure 8) in this case.

As we presented in Section 3, the image features that we use in the leg-based visual servoing are the vectors ${}^{c}{\underline{n}}_{i}^{\left(k\right)}$ (characterizing the line $\ell_{i}$). Thus, we can find the mapping relating the time derivatives of the vectors ${}^{c}{\underline{n}}_{i}^{\left(k\right)}$ to the derivative of $p_{ik}^{\left(1\right)}$, $p_{ik}^{\left(2\right)}$ with respect to time:

(15) $${}^{c}{\dot{\underline{n}}}_{ik}^{\left(k\right)}=J_{nik}[\begin{array}{c} {\dot{p}}_{ik}^{\left(1\right)}\\ {\dot{p}}_{ik}^{\left(2\right)} \end{array}]$$

Then we get the error model,

(16) $$\Delta^{c}{\underline{n}}_{ik}^{\left(k\right)}=J_{nij}[\begin{array}{c} \Delta p_{ik}^{\left(1\right)}\\ \Delta p_{ik}^{\left(2\right)} \end{array}]$$

where $\Delta^{c}{\underline{n}}_{ik}^{\left(k\right)}$, $\Delta p_{ik}^{\left(1\right)}$ and $\Delta p_{ik}^{\left(2\right)}$ are the small variations of the vectors ${}^{c}{\underline{n}}_{ik}^{\left(k\right)}$, $p_{ijk}^{\left(1\right)}$ and $p_{ijk}^{\left(2\right)}$ respectively. Based on the controllers presented in Section 3, for the image features $s={\left[{}^{c}n_{1}^{(1)T}\;\;{}^{c}n_{1}^{(2)T}\ldots {}^{c}n_{n}^{(1)T}\;\;{}^{c}n_{n}^{(2)T}\right]}^{T}$, we have

(17) $$\Delta s=J_{n}\Delta p$$

where Δs stands for the small variations of the image features s, Δp contains all errors $\Delta p_{ik}^{\left(1\right)}$ and $\Delta p_{ik}^{\left(2\right)}$.

In this case, the camera observation noise is set to be ±0.5 pixel, which is a typical noise for cameras. Thus every component of vector $\Delta p_{ik}^{\left(1\right)}$ and $\Delta p_{ik}^{\left(2\right)}$ can take the values +0.5 or −0.5. With the help of Equation (17), we can get the observation error model for LegBVS and LineBVS written under the generic form:

(18) $$\Delta x=L_{P}\Delta p$$

where $L_{P}=L^{+}J_{n}$.

#### 4.2.2. Observation Errors in the Image Moment Visual Servoing

**Figure 9:**
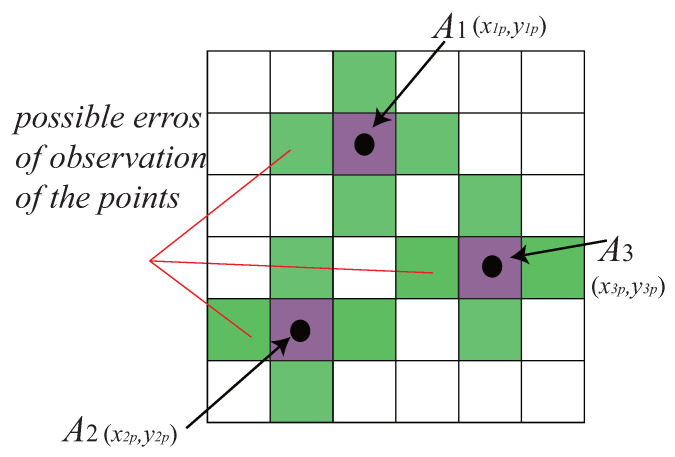
Error on the three points discrete model.

Image moments are calculated from the coordinates of the points belonging to the projection on the image plane of the object observed. We set $\left(x_{1p},y_{1p}\right),\left(x_{2p},y_{2p}\right),\left(x_{3p},y_{3p}\right)$ to be the coordinates of the projection of the three points $A_{1},A_{2},A_{3}$ (Figure 9) in pixel. Then we have

(19) $$\frac{\partial m}{\partial t}=\frac{\partial m}{\partial Q}\frac{\partial Q}{\partial t}=S\frac{\partial Q}{\partial t}$$

where $Q={\left[x_{1p}\;x_{2p}\;x_{3p}\;y_{1p}\;y_{2p}\;y_{3p}\right]}^{T}$ and S is the matrix which transforms the time derivatives of the set of image moments m to the time derivatives of the coordinates of the points projected to the pixel plane.

Thus, Equation (14) can be written as the form

(20) $$\tau =L_{m}^{+}\dot{m}=L_{m}^{+}S\dot{Q}$$

We estimate that the error of estimation of each component of Q to be ±0.5 pix (see Figure 9) for the location of each point projected in the image plane. Then the error model of the image moment visual servoing controller can be written in the form:

(21) $$\Delta x=L_{m}^{+}S\Delta Q$$

#### 4.2.3. Positioning Accuracy

For the Gough–Stewart platform, we have $\Delta x=[\Delta t_{x}\;\Delta t_{y}\;\Delta t_{z}\;\Delta w_{x}\;\Delta w_{y}\;\Delta w_{z}]$ with $\left[\Delta t_{x}\;\Delta t_{y}\;\Delta t_{z}\right]$ being the translation errors along the three axes and $\left[\Delta w_{x},\Delta w_{y},\Delta w_{z}\right]$ being the rotation errors around the three axes. Then the positioning error and the orientation error are defined as in [30]:

(22) $$E_{t}=\sqrt{\Delta t_{x}^{2}+\Delta t_{y}^{2}+\Delta t_{z}^{2}}$$

and the orientation error is defined as

(23) $$E_{w}=\sqrt{\Delta w_{x}^{2}+\Delta w_{y}^{2}+\Delta w_{z}^{2}}$$

In the next section, the optimal design problem for the Gough–Stewart platform will be formulated.

## 5. Optimal Design Procedure

In this section, the design procedure developed in order to obtain the optimal parameters of the Gough–Stewart platform together with the parameters of the controllers are described.

### 5.1. Design Variables

Robot design parameters: As we presented in Section 2, the Gough–Stewart platform can be defined by the following geometric parameters: $r_{a},r_{b},\alpha_{0},\alpha_{1},\alpha_{2}$ (Figure 1c) ($r_{a}=∥\vec{O^{\prime }P_{i}}∥$, $r_{b}=∥\vec{OB_{i}}∥$, $\alpha_{0}=∠x^{\prime }P_{c}P_{0}$, $\alpha_{1}=\frac{1}{2}∠B_{1}B_{c}B_{2}$, $\alpha_{2}=\frac{1}{2}∠P_{1}P_{c}P_{2}$). All these parameters have an effect on the size of robot workspace and the physics performance, as well as on the controller performance. In addition, when LegBVS and LineBVS are applied, the radius of the cylindrical distal links of the Gough–Stewart platform also influence the positioning accuracy [22], thus the radius of the cylindrical distal links $P_{i}B_{i}$ (i=1,2,⋯,6), denoted as *R* (see Figure 3), is a decision variable of the optimization process. When image moment visual servoing is applied, the coordinates of the discrete three points model $\left[x_{1}\;y_{1}\;x_{2}\;y_{2}\;x_{3}\;y_{3}\right]$ (in moving platform frame $x^{\prime }O^{\prime }y^{\prime }$) defining the configuration of the model (Figure 4) affect the controller interaction model. They must be optimized when dealing with image moment visual servoing.

Controller design parameters: The configuration of the camera is normally parameterized by six independent parameters and it affects the controller interaction model. In order to observe the robot (both the robot legs and the end-effector) in a symmetrical way:

- The camera frame orientation is set to be parallel to the robot fixed frame;
- The camera origin is imposed to stay on a vertical line passing through *O* ($\left(x_{c},y_{c}\right)$ of the camera frame origin set at (0,0)).

Additionally, some other variables that we used in the optimal design process need to be defined: *L* is the length of the prismatic actuator $B_{i}P_{i}$ ( $L=∥\vec{B_{i}P_{i}}∥$, i=1,2,⋯,6). $l_{0}$ is the dimensions of the side length of the cube RDW (see Table 1) [1].

Design variables: Based on the explanations above, two different sets of design variables (grouped in a vector y), depending of the types of controllers are defined:

- For the leg-based controllers,
  $y={\left[r_{a},r_{b},\alpha_{0},\alpha_{1},\alpha_{2},z_{c},R\right]}^{T}$;
- For the moment-based controllers,
  $y={\left[r_{a},r_{b},\alpha_{0},\alpha_{1},\alpha_{2},z_{c},x_{1},x_{2},x_{3},y_{1},y_{2},y_{3}\right]}^{T}$.

### 5.2. Objective Function

As mentioned in Section 2, the robot should be as compact as possible. The footprint of the Gough–Stewart platform is evaluated by the radius $r_{b}$ of its base. Therefore, the optimization problem is formulated in order to minimize the value of $r_{b}$.

### 5.3. Constraints

The constraints provided in Section 2 are reviewed here. Throughout the RDW, the following geometric and kinematic constraints must be satisfied:

- The RDW should be free of singularity (both of the robot and the controller): Singularities of the controllers are detailed and presented in Section 4.1. In this case, we used the inverse condition number of the interaction matrix L, denoted as $\kappa^{-1}\left(L\right)$. In the RDW, we want to have
  (24) $$\kappa^{-1}\left(L\right)>10^{-3}$$
  The “mechanics” singularity of the Gough–Stewart platform is different. This problem is complex and was studied decades ago [1,41,42,43,44]. In [45,46], a kinetostatic approach taking account of the force transmission was proposed to determine the singularity-free zones of a parallel robot. When the pressure angle is close to 90 degrees, the parallel robot is close to a singular configuration. Therefore, we calculated the pressure angles $\beta ={\left[\beta_{1},\cdots ,\beta_{6}\right]}^{T}$ for all the six robot legs of the Gough–Stewart platform [45,46]. In the RDW, we want to have
  (25) $$\beta_{i}>80^{\circ }\;i=1,\cdots ,6$$
- The value of the robot positioning accuracy ought to be lower than 1 mm and the orientation accuracy should be lower than 0.02 rad. The positioning error model is defined in Section 4.2. The error models are linear in terms of the observation error, the maximal positioning error $E_{t}\max=\max∥E_{t}∥$ and the maximal orientation error $E_{w}\max=\max∥E_{w}∥$ of the robot will be found at one the corners of the hyper-polyhedron defining the observation errors [47]. The repeatability constraint can be formulated as:
  (26) $$\begin{array}{cc} \; & E_{t}\max\leq 1\;\mathrm{mm}\\ \; & E_{w}\max\leq 0.02\;\mathrm{rad} \end{array}$$
- The discrete three points $A_{1},A_{2},A_{3}$ should be within the moving platform of the Gough–Stewart platform.
- The end-effector should be within the view of the camera: ensuring that all the robot distal legs can be observed when using leg-based visual servoing, as well as the three points $A_{1},A_{2},A_{3}$ can be observed when using image moment visual servoing.
- Several distances or angles are constrained in order to avoid collisions or to have unpractical designs. The values of these constraints are given here:
  (27) $$\begin{array}{cc} \; & 0.4\;m⩽L⩽0.76\;m,\\ \; & 0.1\;m⩽r_{a}⩽0.3\;m⩽r_{b}⩽0.5\;m,\\ \; & r_{a}<0.9\times r_{b},\\ \; & -\frac{\pi }{6}⩽\alpha_{0}⩽\frac{\pi }{6},\\ \; & 0⩽\alpha_{1}⩽\frac{\pi }{9},\\ \; & 0⩽\alpha_{2}⩽\frac{\pi }{4},\\ \; & -0.2\;m⩽z_{c}⩽0.3\;m,\\ \; & 0.01\;m⩽R⩽0.03\;m, \end{array}$$

The aforementioned RDW throughout which all the constraints (24)–(27) must be satisfied should cover a cube of side length $l_{0}\geq 100$ mm and the range of T&T angles being ϕ∈(−π,π], θ∈[0,π/12], and σ∈[0,π/12]. The algorithm of calculating the size of the Largest Regular Dexterous Workspace (LRDW) is detailed and presented in [27] and is adapted in this case for getting the cubic LRDW among the RDW of the manipulator for a given decision variable vector y.

We denote $l_{LRDW}$ the side length of the cubid LRDW whose T&T angles range are ϕ∈(−π,π], θ∈[0,π/12], and σ∈[0,π/12]. We make sure that all constraints (24)–(27) are obligatory true throughout the LRDW, then only one is needed to replace all the other ones, which is defined by:

(28) $$\begin{array}{c} l_{LRDW}\geq 0.1\;m \end{array}$$

### 5.4. Problem Formulation and Optimization Results

For designing a compact Gough–Stewart platform with the detailed specifications given in Table 1, the following optimization problem is formulated:

(29) $$\begin{array}{cc} \mathrm{minimize}\; & r_{b}\\ \mathrm{over}\; & y\\ \mathrm{subject}\;\mathrm{to}\; & l_{LRDW}⩾100\;\mathrm{mm} \end{array}$$

where the definition of y is given in Section 5.1.

As introduced in Section 3, observing three legs is enough to fully control the Gough–Stewart platform when leg-based visual servoing controllers are applied. In this case, as a matter of comparison, we will optimize the geometric parameters of the Gough–Stewart platform when observing only three legs ([Case 1]: observing robot links $B_{1}P_{1}$, $B_{3}P_{3}$, $B_{5}P_{5}$) and observing all the six legs ([Case 2]: observing robot links $B_{1}P_{1}$, $B_{2}P_{2}$, $B_{3}P_{3}$, $B_{4}P_{4}$, $B_{5}P_{5}$, $B_{6}P_{6}$) for leg-based visual servoing.

**Figure 10:**
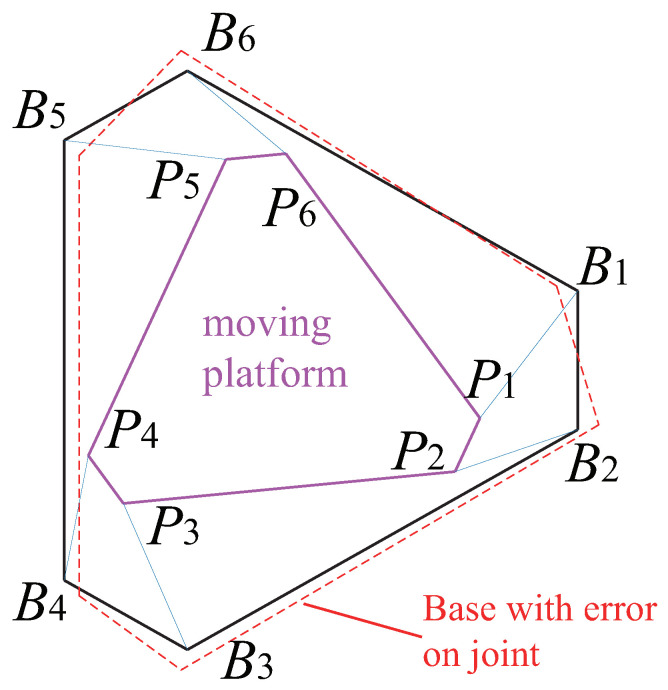
Gough–Stewart platform optimized using LineBVS [Case 1].

**Figure 11:**
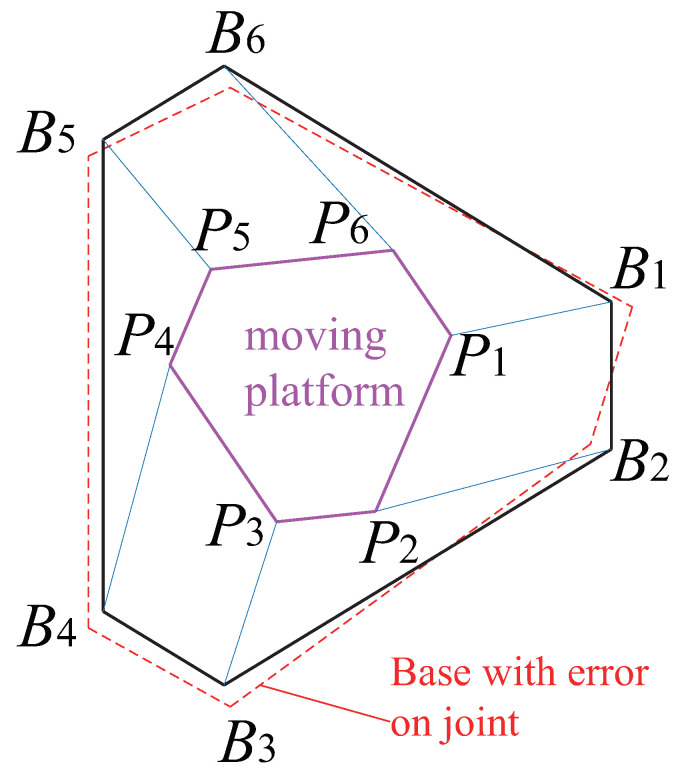
Gough–Stewart platform optimized using LineBVS [Case 2].

**Figure 12:**
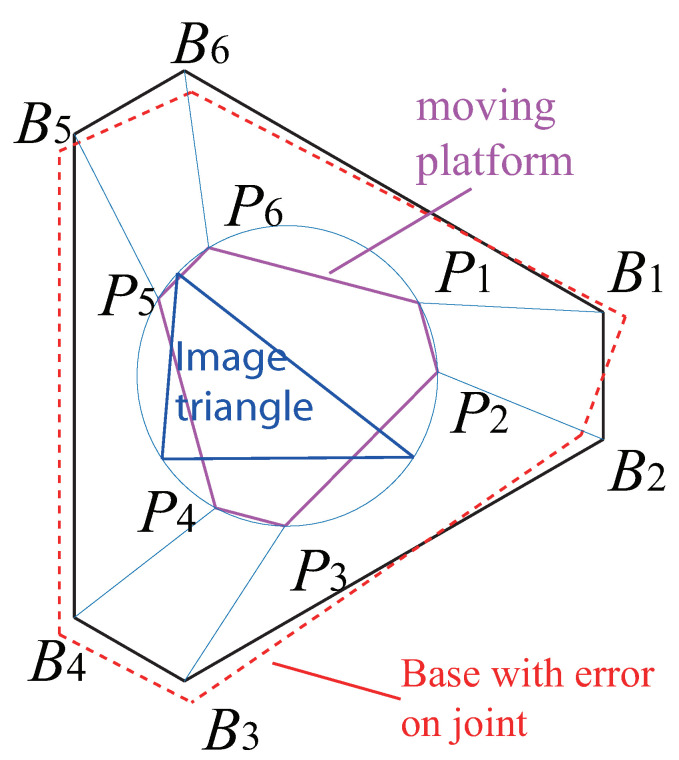
Gough–Stewart platform optimized using image moment visual servoing.

The optimization algorithm presented above is then applied to the design of the Gough–Stewart platform, for each of the three controllers defined in Section 3. These optimization problems have been solved by means of the ‘active-set’ algorithm implemented in the MATLAB *fmincon* function. A *multistart* algorithm, combined with random initial points initialized by a Genetic Algorithm, was also used in order to increase the chances to reach the global minima. The optimal design results are given in Table 2 and illustrated in Figure 10, Figure 11 and Figure 12.

As we see from the results of optimization, in terms of the footprint of the robot, the Gough–Stewart platform designed based on the LegBVS, LineBVS, and IMVS are close from each other and the differences are almost negligible. Especially, for robots designed for leg-based visual servoing controllers, the geometric parameters of the robot are the same under the same observing condition (Case 1 and Case 2). This result proves our hypothesis proposed in Section 4.1.1. The coordinates of points $B_{i}$ are constant since the points $B_{i}$ are fixed, then the time derivative of $h_{i}$ and ${\underline{u}}_{i}$ are linearly dependent, which means that the LegBVS and LineBVS share the same controller performance.

In the next section, we will perform co-simulations with ADAMS and Simulink to test the robot accuracy performance.

## 6. Results Cross-Validations through Simulations

### 6.1. Simulation Method

**Figure 13:**
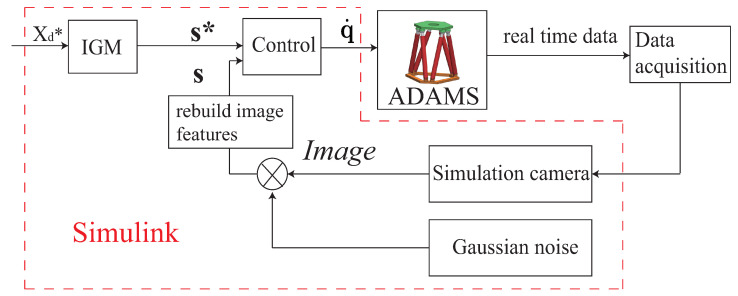
Co-simulation control scheme of Gough–Stewart platform.

In order to validate the optimization results and test the robot accuracy performance, the co-simulations are performed within a connected ADAMS-Simulink environment (Figure 13). Five Gough–Stewart platform models with the optimal geometric parameters obtained from the optimal design process (one model per controller) are created in the software ADAMS.

Real-time data (block “Data acquisition”) of the ADAMS simulator are extracted:

- For LegBVS and LineBVS, we extract the coordinates of the points $P_{i}$ and $B_{i}$ (Figure 1b);
- For IMVS, the coordinates of the three points $A_{1}$, $A_{2}$ and $A_{3}$ (Figure 4) are extracted.

The scheme of the co-simulation is illustrated in Figure 13. The frequency of the simulation is set to be 200 Hz. Real-time data of mechanical models are the output of ADAMS and are sent to Simulink. In Simulink, the model of the camera is created and the real-time data are projected to pixel plane by the camera model to rebuild image features. The ±0.5 pixel random noise related to the observation errors presented in Section 4 is added in the pixel plane. Then the image features with noise become the feedback of the control loop to generate the velocity command. The velocity command is the input of ADAMS and is used to control the motion of the robot mechanical model.

The RDW of the Gough–Stewart platform is a cube whose side length is 100 mm, and the orientation workspace is set based on the T&T angles ϕ∈(−ππ], θ∈[0π/12], σ∈[0π/12]. A home position $T_{1}$ and nine desired positions (including $T_{1}$) within the LRDW are defined in Table 3 with respect to the center of the LRDW. For each position, three orientation pose are defined with respect to ${\left[\phi \;\theta \;\sigma \right]}^{T}$: Pose 1 ${\left[0,0,0\right]}^{T}$, Pose 2 ${\left[\pi /2,\pi /12,\pi /12\right]}^{T}$, and Pose 3 ${\left[-\pi /2,\pi /12,\pi /12\right]}^{T}$. Therefor, for each robot, a total of 27 desired poses are selected in the co-simulations.

Then, each robot is driven from their home pose to the desired poses with the dedicated controller. All their positioning accuracies and orientation accuracies are recorded during the co-simulation.

Additionally, in order to test the robustness of the accuracy of model with geometry errors, the same co-simulations were operated with the error added in model. The models we added errors on joints to are defined as below: we add a random error on the location of the joint $B_{i}$ on the base of the robot, the distance between the accurate joint $B_{i}$, and the joint with error $B_{i}^{\prime }$, denoted as $l_{B_{i}B_{i}^{\prime }}$, ($l_{B_{i}B_{i}^{\prime }}=0.1\times r_{b}$) (see the red parts of Figure 10, Figure 11 and Figure 12).

In the next step, The designed robot prototypes were controlled with another controller, different from the one dedicated during the design process, for verifying the original purpose of performing control-based design. In what follows, for brevity, only the result of LineBVS applied to the robot designed for the image-based moments will be given here.

Results are shown and analyzed in the next subsection.

### 6.2. Simulation Results

In this section, we denote as:

- [Case A]: the Gough–Stewart platform optimized for LineBVS ([Model 1]) in ([Case 1]) and the error-added robot mechanical model ([Model 2]) controlled with their dedicated controller, robot links $B_{i}P_{i}$ (i=1,3,5) are observed;
- [Case B]: the Gough–Stewart platform optimized for LineBVS ([Model 3]) in ([Case 2]) and the error-added robot mechanical model ([Model 4]) controlled with their dedicated controller, all six robot links $B_{i}P_{i}$ (i=1,2,3,4,5,6) are observed;
- [Case C]: the Gough–Stewart platform optimized for IMVS ([Model 5]) and the error-added robot mechanical model ([Model 6]) controlled with its dedicated controller;
- [Case D]: the Gough–Stewart platform optimized for IMVS ([Model 5]) controlled with the LineBVS, all six links $B_{i}P_{i}$ (i=1,2,3,4,5,6) are observed.
- [Case E]: the Gough–Stewart platform optimized for LineBVS ([Model 3]) controlled with the IMVS.

Since we have proved that the LegBVS and LineBVS have the same control performance for the Gough–Stewart platform and the geometric parameters of the robot designed for these two controllers are the same under the same observation condition, we only perform the co-simulations for the robot that controlled with LineBVS. We played each simulation for five seconds and recorded the positioning error. The simulation results show that the robot converges at around 0.5 s, then the moving platform oscillates around the desired pose due to the simulated observation noise. For all the simulation motions from home position to the desired poses in Case A to Case E, the maximal positioning error and orientation error along the time are recorded: for point $T_{kj}$ (*k* for the position k=1,…,9, *j* for the pose j=1,2,3) simulated in case α (α=A,B,C,D), this maximal positioning error is denoted as $\delta p_{kj\alpha }$ and the maximal orientation error is denoted as $\delta o_{kj\alpha }$. Then, all the results are summarized in Table 4: for each case and different model, max, min, and mean value of positioning error $\delta p_{kj\alpha }$ and orientation error $\delta o_{kj\alpha }$ obtained for k=1,…,9 and j=1,2,3 are shown for a given value of α.

Studying the results, we see that the robot Model 5 in Case C leads to minimal positioning error and orientation error. For robots in Case A and Case B, the mean value is very close to the requested value of 1 mm. However, there are some points in the workspace for which the error is slightly upper this limit (maximal error of 1.24 mm in both cases). In fact, the positioning accuracy model applied during the optimal design process (Section 5) in order to estimate the controller performance was really simple. It was thus the source of inaccuracies of positioning error estimation during the optimal design process. However, even with this simplistic model, the maximal robot positioning error (1.24 mm) is only slightly upper the threshold of 1 mm while their mean values stay close to 1 mm. Additionally, the measured orientation error obtained from all the cases are far lower than the requested 0.01 rad. The results obtained from the models with geometry errors are similar to the results obtained from the accurate models, which proves the robustness of the accuracy of models when applying the visual servoing controllers.

We then study the results of [Case D] and [Case E], which are the most important. For the Gough–Stewart platform optimized for IMVS but controlled with the LineBVS, the mean error is far bigger than the requested value of 1 mm, and the maximal error even grows up to 1.56 mm. For the Gough–Stewart platform optimized for LineBVS but controlled with the IMVS, the mean error is 1.39 mm, and the maximal error grows to 1.47 mm. These positioning errors are bigger than the requested value of 1 mm and they are worse than the results of [Case B] and [Case C]. These results confirm that it is necessary to optimize a robot for a dedicated controller. In other words, the control-based design of {robot+controller} helps ensure the vision-based control accuracy performance.

**Figure 14:**
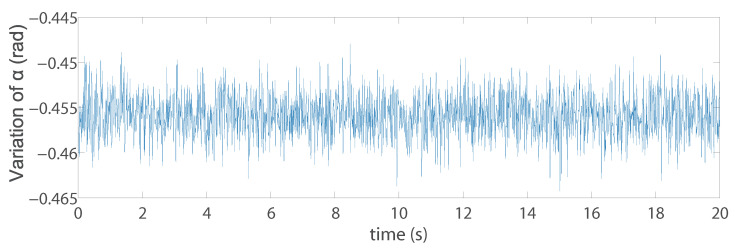
Variation of the image moment α for the Triangle 1.

**Figure 15:**
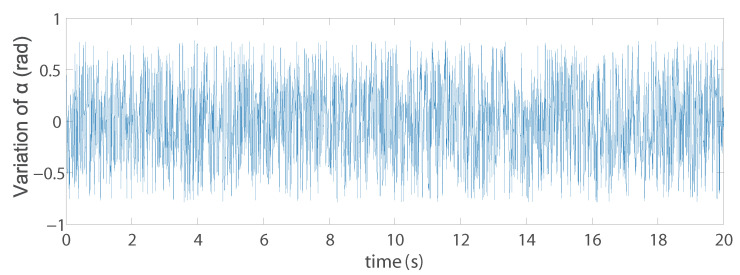
Variation of the image moment α for the Triangle 2.

Another problem, which is the most interesting, is that the discrete three points model we obtained from the optimal design when using IMVS to form a triangle (Triangle 1) which is not a regular triangle. Therefore, in order to study why it is such a configuration, we create discrete three points whose configuration is a regular triangle (Triangle 2). The coordinates of the three points (with respect to the moving platform frame $x^{\prime }O^{\prime }y^{\prime }$) $A_{1r},A_{2r},A_{3r}$ are (0, 0.222) m, (0.192, −0.111) m, and (−0.192, 0.111) m. The same noise was added on the projection of the points in pixel to see the variation of the set of image moments m. For Triangle 1 and Triangle 2, in terms of the image moments $\left[x_{g},y_{g},a\right]$, the variations are almost the same. However, in terms of the image moments $\left[\alpha ,c_{1},c_{2}\right]$, the differences are huge: for Triangle 1, the variations of $\left[\alpha ,c_{1},c_{2}\right]$ are [0.01,0.08,0.08], while the variations of the image moments $\left[\alpha ,c_{1},c_{2}\right]$ for Triangle 2 are [1.6,20,400] (the results of α are illustrated in Figure 14 and Figure 15). In addition, we performed the same co-simulation as we did for Model 5 in Case C, but the target is changed to the new three discrete points model (Triangle 2) in IMVS. The simulation results show that the maximal positioning error comes to 1.6 mm and the maximal orientation error comes to 6.0 × 10${}^{-4}$ rad, while the corresponding results are 0.63 mm and 4.3 × 10${}^{-4}$ rad when observing the three discrete points model Triangle 1. The results prove that the configuration of the discrete points model has an influence on the observation of image moments and affects the controller accuracy performance. As a result, it is necessary to perform topology optimization on the configuration of the shape of the target observed during the design process.

## 7. Conclusions

In the work presented above, a novel advanced optimal design methodology “control-based design” is performed in order to design a Gough–Stewart robot with the best accuracy performance of the pair {robot + controller}. We have proven that the controller performance (accuracy, singularity) is affected by the robot geometric design parameters. Thus, in the design process of a robot, it is necessary to find the optimized geometric parameters of the robot that will allow the best performance of the pair {robot + controller}.

Three different classical types of visual servoing controllers: LegBVS, LineBVS, and IMVS were proposed to be applied on the Gough–Stewart platform. Positioning error models considering the camera observation error were developed based on the study of these three types of controllers. In addition, in order to avoid the instability issues, the singularities of these controllers were analyzed for purpose of avoiding the controller singularities. In the next step, the design optimization problem for getting the optimal geometric parameters and the placement of the camera for the Gough–Stewart platform has been formulated for each type of controller. Then, co-simulations between ADAMS and Simulink for the Gough–Stewart platforms optimized for the three controllers were performed. The results showed that the robots designed for these three visual servoing controllers had a similar size (robots designed for LegBVS and LineBVS share the same size). The robot designed for IMVS had a better positioning accuracy compared with the other two robots optimized for LegBVS and LineBVS. Especially, the co-simulation results show that when one controller is applied on a robot designed for another one, the positioning error performance was no longer guaranteed, confirming the importance of the control-based design approach. In the future, experimental works on real prototypes are necessary in order to verify the simulation results.

## Figures and Tables

**Table 1:** Requirements of the Gough–Stewart platform.

Cube RDW size (side length of the cube) $l_{0}$	⩾100 mm
Tilt and Torsion angles	ϕ∈(−π,π], θ∈[0,π/12], σ∈[0,π/12]
Positioning accuracy in RDW	⩽1 mm
Orientation accuracy in RDW	⩽0.01 rad
No singularity in RDW	of the controller of the robot
Constraints on geom. param.	will be provided in Section 5

**Table 2:** Design parameters and value of the objective function for the chosen controller.

	LegBVS	LineBVS	LegBVS	LineBVS	IMVS
	[Case 1]	[Case 1]	[Case 2]	[Case 2]	
$r_{a}\left[m\right]$	0.2054	0.2054	0.1402	0.1402	0.1600
$r_{b}\left[m\right]$	0.3000	0.3000	0.3000	0.3000	0.3000
$\alpha_{0}\left[\mathrm{rad}\right]$	−0.4256	−0.4256	−0.4243	−0.4243	0.2668
$\alpha_{1}\left[\mathrm{rad}\right]$	0.2318	0.2318	0.2298	0.2298	0.1986
$\alpha_{2}\left[\mathrm{rad}\right]$	0.1424	0.1424	0.6927	0.6927	0.2406
$z_{c}\left[m\right]$	−0.0523	−0.0523	−0.0551	−0.0551	0.1204
R [m]	0.0197	0.0197	0.0199	0.0199	N/A
$x_{1}\left[m\right]$	N/A	N/A	N/A	N/A	−0.1311
$x_{2}\left[m\right]$	N/A	N/A	N/A	N/A	0.1303
$x_{3}\left[m\right]$	N/A	N/A	N/A	N/A	−0.1044
$y_{1}\left[m\right]$	N/A	N/A	N/A	N/A	−0.0870
$y_{2}\left[m\right]$	N/A	N/A	N/A	N/A	−0.0839
$y_{3}\left[m\right]$	N/A	N/A	N/A	N/A	0.0976
$r_{b}\left[m\right]$	0.3000	0.3000	0.3000	0.3000	0.3000

**Table 3:** Coordinates of the test points parameterized with respect to the center of the LRDW.

Point	Coordinate [m]	Point	Coordinate [m]	Point	Coordinate [m]
$T_{1}$	(0,0,0)	$T_{4}$	(−0.05,−0.05,0.05)	$T_{7}$	(0.05,0.05,−0.05)
$T_{2}$	(0.05,0.05,0.05)	$T_{5}$	(−0.05,0.05,0.05)	$T_{8}$	(0.05,−0.05,−0.05)
$T_{3}$	(0.05,−0.05,0.05)	$T_{6}$	(−0.05,0.05,−0.05)	$T_{9}$	(−0.05,−0.05,−0.05)

**Table 4:** Results of co-simulation in terms of end-effector accuracy: min, max, standard deviation, and mean values for the error recorded on the tested 24 points.

Case	Max Positioning Error [mm]	Min Positioning Error [mm]	Mean Positioning Error [mm]	Max Orientation Error [rad]	Min Orientation Error [rad]	Mean Positioning Error [rad]
A ([Model 1])	1.24	0.94	1.03	4.5 × 10${}^{-4}$	2.2 × 10${}^{-4}$	3.1 × 10${}^{-4}$
A ([Model 2])	1.23	0.96	1.05	4.5 × 10${}^{-4}$	2.0 × 10${}^{-4}$	3.5 × 10${}^{-4}$
B ([Model 3])	1.12	0.91	0.99	4.0 × 10${}^{-4}$	1.9 × 10${}^{-4}$	2.8 × 10${}^{-4}$
B ([Model 4])	1.24	0.99	1.01	4.0 × 10${}^{-4}$	2.1 × 10${}^{-4}$	3.0 × 10${}^{-4}$
C ([Model 5])	0.63	0.28	0.38	4.3 × 10${}^{-4}$	2.2 × 10${}^{-4}$	2.8 × 10${}^{-4}$
C ([Model 6])	0.66	0.29	0.42	4.6 × 10${}^{-4}$	2.6 × 10${}^{-4}$	3.0 × 10${}^{-4}$
D ([Model 5])	1.56	1.37	1.44	5.0 × 10${}^{-4}$	3.3 × 10${}^{-4}$	4.4 × 10${}^{-4}$
E ([Model 5])	1.47	1.21	1.39	4.3 × 10${}^{-4}$	2.3 × 10${}^{-4}$	3.4 × 10${}^{-4}$

## Data Availability

Not applicable.

## Appendix A. Leg-Based Visual Servoing Interaction Matrix

### Appendix A.1. Leg-Direction-Based Visual Servoing Interaction Matrix

Leg-direction-based visual servoing kinematic aims at finding the relationship between the time variation of the unit vector ${\underline{u}}_{i}$ of the robot legs and the twist of its platform.

The leg direction ${\underline{u}}_{i}$ extracted from the observation of the robot leg $B_{i}P_{i}$ (Figure 1b) is selected as the feature to do the visual servoing. We have

(A1) $${\underline{u}}_{i}=\left(P_{i}^{G}-B_{i}^{G}\right)/L_{i}$$

where $L_{i}$ is the length of the prismatic actuator $B_{i}P_{i}$. $P_{i}^{G}$ and $B_{i}^{G}$ represent the coordinates with respect to the global frame. $P_{i}^{G}$ (i=1⋯6) can be obtained from:

(A2) $$P_{i}^{G}=T+R\left(\phi ,\theta ,\sigma \right)P_{i}^{L}\;\;\;\;\;\;\;\;\;\;\;\left(i=1\cdots 6\right)$$

T and R denote the position of the center of the mobile frame and its rotation matrix in the global frame. $P_{i}^{L}$ denotes the coordinate of the point $P_{i}$ in local frame.

From Equation (A2), we have the vision-based kinematics of the Gough–Stewart platform expressed in the global frame:

(A3) $$L_{i}{\underline{u}}_{i}=T+R\left(\phi ,\theta ,\sigma \right)P_{i}^{L}-B_{i}^{G}$$

(A4) $${\underline{\dot{u}}}_{i}=\frac{1}{L_{i}}\frac{1}{dt}\vec{P_{i}B_{i}}-\frac{{\dot{L}}_{i}}{L_{i}}{\underline{u}}_{i}$$

By inserting the interaction matrix associated to a 3D point [16], we get

(A5) $${\underline{\dot{u}}}_{i}=-\frac{1}{L_{i}}\left[I_{3}-{\left[B_{i}\right]}_{\times }\right]\tau -\frac{{\dot{L}}_{i}}{L_{i}}{\underline{u}}_{i}$$

where ${\left[\cdots \right]}_{\times }$ is the antisymmetric matrix associated to the cross product [16]. With the help of the inverse Jacobian matrix, we can obtain the relationship between each ${\dot{\underline{u}}}_{i}$ and τ.

(A6) $${\dot{\underline{u}}}_{i}=M_{ui}^{T}\tau$$

(A7) $$M_{ui}^{T}=-\frac{1}{L_{i}}\left(I_{3}-{\underline{u}}_{i}{\underline{u}}_{i}^{T}\right)\left[I_{3}-{\left[B_{i}\right]}_{\times }\right]$$

Then, the interaction matrix $M_{u}^{T}$ can be obtained by stacking the matrices $M_{ui}^{T}$ of k legs (k = 3, 4, 5, 6).

### Appendix A.2. Line-Based Visual Servoing Interaction Matrix

Line-based visual servoing kinematic aims at finding the relationship between the time variation of the Plücker coordinates $\left({\underline{u}}_{i},h_{i}\right)$ of the robot legs and the twist of its platform.

From the definition of the Plücker coordinates, we have $h_{i}=D\times {\underline{u}}_{i}$ where D is the position of any point on the line passing through the center of the cylindrical leg. Then we have

(A8) $${\dot{h}}_{i}=\dot{D}\times {\underline{u}}_{i}+D\times {\dot{\underline{u}}}_{i}$$

For the Gough–Stewart platform, the U joints $B_{i}$(i=1,2⋯6) (Figure 1b) are all fixed on the base platform, then the Equation (A8) can be written in the form

(A9) $${\dot{h}}_{i}={\dot{B}}_{i}\times {\underline{u}}_{i}+B_{i}\times {\dot{\underline{u}}}_{i}=B_{i}\times {\dot{\underline{u}}}_{i}$$

With the help of Equation (A6), the Equation (A9) can be written in the matrix form

(A10) $${\dot{h}}_{i}={\left[B_{i}\right]}_{\times }\times M_{ui}^{T}\tau =M_{hi}^{T}\tau$$

$M_{i}$ is the interaction matrix related to the vector ${\underline{u}}_{i}$.

Therefore, for a line $L_{i}$, we have

(A11) $$[\begin{array}{c} {\dot{\underline{u}}}_{i}\\ {\dot{h}}_{i} \end{array}]=[\begin{array}{c} M_{ui}^{T}\\ M_{hi}^{T} \end{array}]\tau =M_{uhi}^{T}\tau$$

Then, the interaction matrix $M_{uh}$ can be obtained by stacking the matrices of $M_{uhi}^{T}$ of k legs (k = 3, 4, 5, 6).
